# Bleeding risk stratification in coronary artery surgery: the should-not-bleed score

**DOI:** 10.1186/s13019-021-01473-3

**Published:** 2021-04-21

**Authors:** Mirna Petricevic, Mate Petricevic, Marijan Pasalic, Branka Golubic Cepulic, Mirela Raos, Vesna Vasicek, Klaus Goerlinger, Kresimir Rotim, Hrvoje Gasparovic, Bojan Biocina

**Affiliations:** 1 Department of Cardiac Surgery, University Hospital Center Zagreb, Zagreb, Croatia; 2 University Department of Health Studies, University of Split, Split, Croatia; 3 Department of Cardiac Surgery, University Hospital Center Zagreb – Rebro, Zagreb, Croatia; 4 Department for Cardiovascular Diseases, University Hospital Center Zagreb, Zagreb, Croatia; 5 Clinical Department of Transfusion Medicine and Transplantation Biology, University Hospital Centre Zagreb, Zagreb, Croatia; 6 Accounting Department, University of Zagreb Faculty of Economics and Business, Zagreb, Croatia; 7 Klinik fur Anästhesiologie und Intensivmedizin, Universitätsklinikum Essen, Universität Duisburg-Essen, and TEM International GmbH, Munich, Germany; 8 University of Applied Health Sciences, Zagreb, Croatia

**Keywords:** Coronary artery bypass grafting, Bleeding, Transfusion, Bleeding risk score

## Abstract

**Background:**

An estimated 20% of allogeneic blood transfusions in the United States are associated with cardiac surgery. It is estimated that 11% of red cell resources were used for transfusion support of patients undergoing coronary artery bypass grafting (CABG) with a documented wide variability in transfusion rate (7.8 to 92.8%). To address the issue of unnecessary transfusions within the CABG population, we developed a model to predict which patients are at low risk of bleeding for whom transfusion treatment might be considered unnecessary. Herein we present our “SHOULD-NOT-BLEED-SCORE” application developed for the Windows® software platform which is based on our previous research.

**Methods:**

This study is aimed to develop a user-friendly application that stratifies patients with respect to bleeding risk. The statistical model we used in our previous research was focused on detection of CABG patients at low risk of bleeding. The rationale behind such an approach was to identify a CABG patient subgroup at low risk of bleeding. By identifying patients at low risk of bleeding we can define a subgroup of patients for whom transfusion treatment might be considered unnecessary. We developed a Windows platform application based on risk modelling which we previously calculated for 1426 patients undergoing elective CABG from January 2010 to January 2018.

**Results:**

The SHOULD-NOT-BLEED-SCORE risk score is developed for the Windows software platform. A mathematical model that is based on multivariate analysis was used for app development. The variables that entered the scoring system were: Age; Body Mass Index; Chronic Renal Failure; Preoperative Clopidogrel Exposure; Preoperative Red Blood Cells Count; Preoperative Fibrinogen Level; Preoperative Multiplate ASPI test area under the curve (AUC) units. The SHOULD-NOT-BLEED-SCORE identifies/predicts patients without a risk for excessive bleeding with strong discriminatory performance (Receiver Operating Curve (ROC) analysis AUC 72.3%, *p* < 0.001).

**Conclusion:**

The SHOULD-NOT-BLEED risk scoring application may be useful in the preoperative risk screening process. The clinical and economic burden associated with unnecessary transfusions may be adequately addressed by a preoperative scoring system detecting patients at low risk of bleeding for whom transfusion treatment might be considered unnecessary.

## Background

Cardiac surgery is associated with significant perioperative bleeding and carries very high risk for transfusion of allogeneic blood components.

The association between severe postoperative bleeding and poor outcomes has already been widely described in the literature [1–3]. The same association is present between transfusion treatment and poor ouotcomes.

Recently, Vlot et al. came out with the study analyzing intra-operative red blood cell transfusion and mortality after cardiac surgery [4]. Intra-operative transfusion was associated with a more than three-fold increased risk of 30-day mortality [4]. The association between intra-operative transfusion and mortality persisted after adjustment for known risk factors (adjusted OR 2.6; *P* = 0.007) [4]. Authors concluded that intra-operative transfusion of red blood cells (RBC) was found to be associated with increased mortality in adults undergoing CABG [4].

Hua Chan et al. provided similar findings in their recent study [5]. In their study, perioperative RBC transfusion in isolated CABG patients was associated with increased risks of developing adverse events such as prolonged ventilatory support, cardiac morbidity, renal morbidity and serious infection [5]. It is not just RBC transfusion per se that carries a significant risk for morbidity and mortality in CABG patients. The amount of transfusion remains to be very important. Koch et al. investigated morbidity and mortality risk associated with RBC and blood-component transfusion in isolated CABG [6]. Perioperative RBC transfusion was found to be the single factor most reliably associated with increased risk of postoperative morbid events after isolated CABG [6]. Moreover, each RBC unit transfused was associated with incrementally increased risk for adverse outcome [6].

Transfusion of blood components in CABG patients is primarily empiric resulting in an inevitably wide variability in transfusion rates between different cardiac surgery centers (range 7.8 to 92.8%) [7]. This, in turn, results in a significant economic burden and unnecessary expenditures, be it from transfusion costs or costs related to transfusion associated complications, as well as overhead expenses [8, 9].

When it comes to the haemostatic management, the focus of many researchers, including our research team was to identify the patients at high risk of bleeding who might benefit from more aggressive and more targeted haemostatic management. In patients considered to be at high risk of bleeding, the aim of haemostatic management is to provide targeted and efficient haemostatic treatment (mostly procoagulant blood components). The idea is to avoid transfusions whenever possible and to reduce transfusion requirements with targeted, efficient transfusions.

However, high prevalence (up to 92.8%) of transfusions in patients undergoing isolated CABG, considered as lowest risk cardio-surgical procedure, raises the question to which extent those transfusions were unnecessary. Goodnough et al. investigated costs of blood support given to patients undergoing CABG along with costs of blood components whose transfusions were identified to be unnecessary. The number of blood components transfused unnecessarily represented up to 43% of all blood units transfused. The percentage of total blood costs that was incurred by inappropriate transfusions among institutions was found to be as high as 44% [10]. Identification of patients at high risk of bleeding and shifting their haemostatic management towards more efficient and targeted transfusion treatment presents, however, only one way to reduce unnecessary transfusions.

The other way to reduce the clinical and economic burden of unnecessary transfusions is to completely avoid transfusions in patients undergoing low risk procedures such as CABG, particularly, if the patients are considered to be at low risk of bleeding at the same time. Recently, we changed our paradigm of haemostatic management and shifted our focus towards identification of low bleeding risk patients undergoing low risk procedures (i.e. CABG) [8]. Herein we present our institutional bleeding risk score, based on our own data and own, recently published, peer reviewed results [8]. The score was embedded into a Windows platform application with user friendly interface and was named “SHOULD-NOT-BLEED” score. The main goal of this score is to identify a group of patients with estimated low risk of bleeding undergoing low risk cardio-surgical procedure such as CABG, for whom transfusion treatment might be considered unnecessary.

To the best of our knowledge, this is the first time we have the application/software developed for the bleeding risk scoring.

## Methods

This study is designed as a proof-of-concept study (non-interventional) study.

We developed a SHOULD-NOT-BLEED-SCORE Windows platform application. The application is based on the results of our recent study [8].

The SHOULD-NOT-BLEED-SCORE risk scoring tool was developed in collaboration between University of Split, University Department of Health Studies, University of Split School of Medicine, University of Zagreb - Department of Cardiac Surgery, University of Zagreb - Department of Cardiovascular Diseases, University of Zagreb - Faculty of Economics and Business, University of Applied Health Sciences, University of Zagreb - Division for transfusion medicine.

### Ethical approval

The SHOULD-NOT-BLEED-SCORE risk scoring tool is based on our previous research data in a paper that has already been published [8]. The intention to develop a scoring system based on published data for which we already have approval was expressed to the Institutional Ethics committee. The Institutional Ethics committee approved the study and given the retrospective nature of the study informed written consent was waived.

### Data retrieval

This scoring system is based on 1426 consecutive patients undergoing elective isolated CABG from January 2010 to January 2018.

The premise behind this scoring system is that the transfusion of different blood products in low-bleeding patients was nonessential, resulting in both unwanted consumption of blood products and unnecessary financial costs. Therefore, all blood products given to low bleeding risk patients, as well as their cost, were deemed inappropriate and were used to determine the „saving potential“, i.e. the number and the price of blood products that can be spared if not used for unnecessary treatment in patients with low bleeding risk.

Following an extensive univariate analysis, multivariable binary logistic regression was utilized to create models predicting low bleeding risk CABG patients (Table 1). Initially created with the aim to identify patients without excessive bleeding, the developed model had a specificity as high as 94% whereas sensitivity was 24%. This is in line with the study premise necessitating clear division of high bleeding risk patients receiving necessary blood transfusion and low bleeding risk patients for whom transfusion treatment might be considered unnecessary.

**Table 1:** Multivariate logistic regression model used as a mathematical platform for SHOULD-NOT-BLEED score calculator

		Exp(B)	95% C.I.for EXP(B)		Sig.
			Lower	Upper	
	Age (Years)	0,972	0,957	0,988	0,001
	Body Mass Index	1170	1129	1213	< 0,001
	Chronic Renal Failure	0,541	0,299	0,979	0,042
	Clopidogrel Exposure	0,627	0,463	0,850	0,003
	Calcium Channel Blockers	1371	1050	1788	0,020
	ACE inhibitors	1393	1053	1842	0,020
	Red Blood Cells Count	1319	1015	1715	0,039
	Fibrinogen	1321	1162	1503	< 0,001
	ASPI Multiplate aggregometry test (AUC)	1010	1005	1016	< 0,001
	Constant	0,012			< 0,001

## Results

In our previous and already published research [8], we performed a retrospective observational study on patients undergoing CABG with the aim to define nonessential transfusions resulting in unnecessary financial costs and unwanted consumption of blood products [8]. 1426 patients were included, with their demographic, laboratory, and surgical parameters being collected. Study outcomes included the extent of perioperative bleeding and consequential transfusion rates.

Descriptive analysis of the population gave an insight into the patient’s clinical, laboratory and surgical characteristics. As a part of the outcome analysis, the magnitude of postoperative bleeding, transfusion rates and financial cost of the entire patient population were also assessed. According to the magnitude of the postoperative bleeding, patients were classified into two groups: excessive-bleeding patients, defined as those exhibiting a blood loss within the upper quartile of the patient population (11.33 mL/kg or more, a value shown in our previous research [11]), and non-excessive bleeding patients, those not exhibiting the aforementioned blood loss.

As a part of our previous study and its aim, patient groups were compared according to transfusion rates and the consequential financial costs [8]. Normality of distribution was assessed using the Kolmogorov-Smirnov test while also plotting the distribution and accounting for skewness and kurtosis of the sample. Intergroup comparison was done using the Student t test and Mann Whitney U test.

The premise of the study was that the transfusion of different blood products in non-excessive bleeding patients was nonessential and resulted in both unwanted consumption of a limited asset and unnecessary financial costs [8].

In order to determine the differences between excessive and non-excessive bleeding patients, a group comparison was performed using the Student t test, Mann Whitney U test and χ2 test. Patients were compared across a wide variety of parameters representing their demographic, clinical, laboratory, and surgical characteristics. Patient bleeding risk in our study was defined as an affiliation with the excessive-bleeding group. Correlation analysis (using Spearman correlation coefficient) was performed to identify the potential predictors of bleeding risk. Following an extensive univariate analysis, multivariable binary logistic regression was utilized to create models predicting bleeding risk in a patient undergoing CABG [8].

**Fig. 1:**
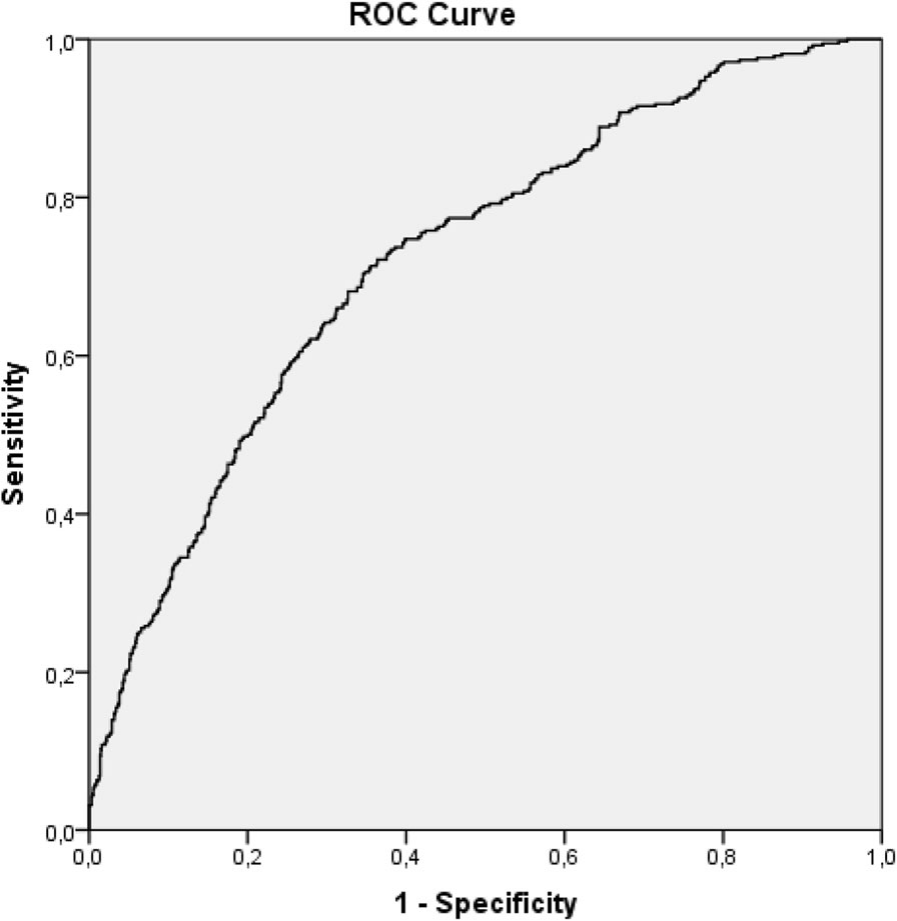
The receiver operating curve (ROC) analysis tested the accuracy of the multivariate logistic regression model. (AUC 0.723 (0,694–0.753), *p* < 0.001)

The accuracy of the generated model was evaluated using ROC analysis (Fig. 1). Finally, based on the model’s accuracy, a “real-life” saving potential was calculated. (expressed both as the number and cost of blood products).

Using the most accurate model and its binary logistic regression equation, a SHOULD-NOT-BLEED-SCORE was generated. The score itself incorporated nine independent parameters, which were previously identified as having the strongest association with bleeding outcome [8]. These parameters included five scalar (measurement) and four nominal (group) variables. Scalar parameters were as follows: patient age, patient body mass index, preoperative red blood cell count, preoperative serum fibrinogen levels and preoperative Multiplate® ASPI values. Nominal parameters were the following: existence of preoperative chronic renal failure, preoperative clopidogrel therapy and preoperative arterial hypertension associated with calcium channel blockers or ACE inhibitor use [8].

The equation output stands for the probability of a patient with the given factors being a high bleeder according to the study definition. The model output was further stratified into four risk groups according to the quartile ranges of the regression equation results in our patient population. Risk groups are assigned with the following titles: low probability, medium probability, medium-high probability, and high probability.

Finally, an application based on the new bleeding risk score was created for Microsoft Windows Platform. In order to achieve a simple input and clear result representation, the graphical user interface was programmed with a “user friendly” intention in mind. Application input parameters stand for the previously mentioned most probable patient outcome predictors. Patient age, patient body mass index (measured in kg/m2), preoperative red blood cell count (measured as N × 10^12^ per liter), preoperative fibrinogen serum levels (measured in grams per liter) and ASPI aggregation values (measured in aggregation units) have to be entered in exact number form. The presence of the chronic renal failure, recent clopidogrel use, and arterial hypertension associated with calcium channel blockers or ACE inhibitor use need to be assessed as well. The SHOULD-NOT-BLEED-SCORE result is expressed both as an absolute risk value and as risk group allocation. We created a color code system to sort patients into their respective risk groups: low probability – green, medium probability – yellow, medium-high probability – maroon, high probability - red.

A two-tailed *p* value < 0.05 was considered to be statistically significant for all deployed calculations, while additional one-tailed *p* values were also obtained and provided for comparisons likely to result in one-directional relationships. Analysis was performed using the IBM SPSS Statistics software package (version 21). The application was developed using the Embarcadero RAD Studio software development package (XE5 version).

**Fig. 2:**
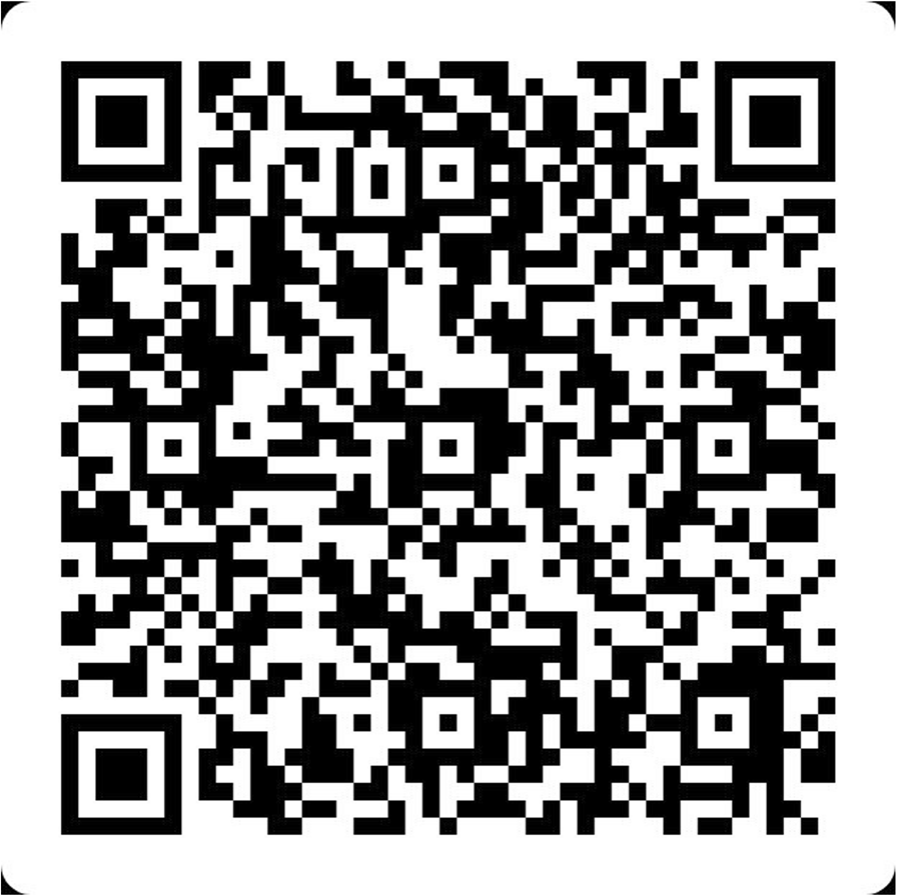
QR code (URL link to download the SHOULD-NOT-BLEED-SCORE Windows platform application)

The URL web link to download the SHOULD-NOT-BLEED-SCORE application is shown using a QR code in Fig. 2. The QR code leads the user directly to the application stored on the cloud and may be downloaded free of charge. The user interface is user friendly and self-explanatory so any user may easily approach and calculate bleeding risk. SHOULD-NOT-BLEED-SCORE provides an exact percentage for bleeding risk coupled with the color code previously described. Herein we provide two examples of calculations.

### Case scenario 1

**Fig. 3:**
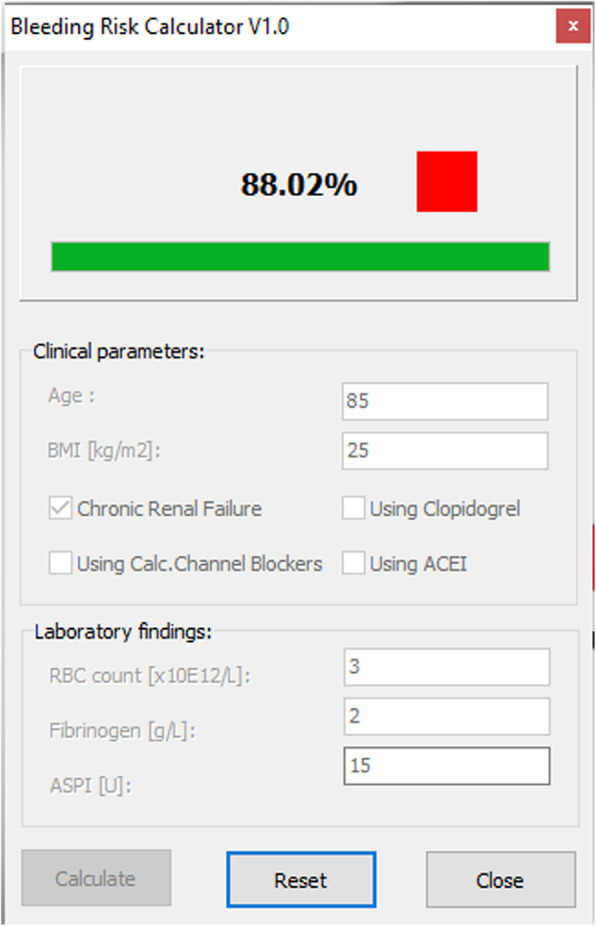
Case scenario 1

An 85 year old patient presents to the emergency department with chest pain and shortness of breath. Diagnostic coronary angiography shows severe left main stenosis. The patient is on dual antiplatelet therapy (Aspirin 100 mg + clopidogrel 75 mg) given their history of vascular intervention on the right lower limb. The patient has known renal failure, BMI of 25 kg/m2, RBC count 3 × 10^12^, Fibrinogen count of 2 g/L and a Multiplate® ASPItest value of 15 AUC units (Fig. 3). SHOULD-NOT-BLEED-SCORE calculates bleeding risk to be 88.02% coupled with a red colored square suggesting high risk of bleeding.

### Case scenario 1 interpretation

This patient has a high risk for excessive bleeding. Some of the SHOULD-NOT-BLEED-SCORE parameters contributing to the risk of bleeding are invariable (*ie.* age and renal function) whereas some other parameters are modifiable (*ie.* waiting time following clopidogrel cessation, Aspirin cessation given the pronounced platelet inhibition, as assessed by Multiplate ASPI test, and management of preoperative anemia (1st pillar of the patient blood management)). The SHOULD-NOT-BLEED-SCORE is not developed to guide the clinical decision-making process, but rather as a useful tool to stratify risk of bleeding and to point out factors contributing to existing risk.

### Case scenario 2

**Fig. 4:**
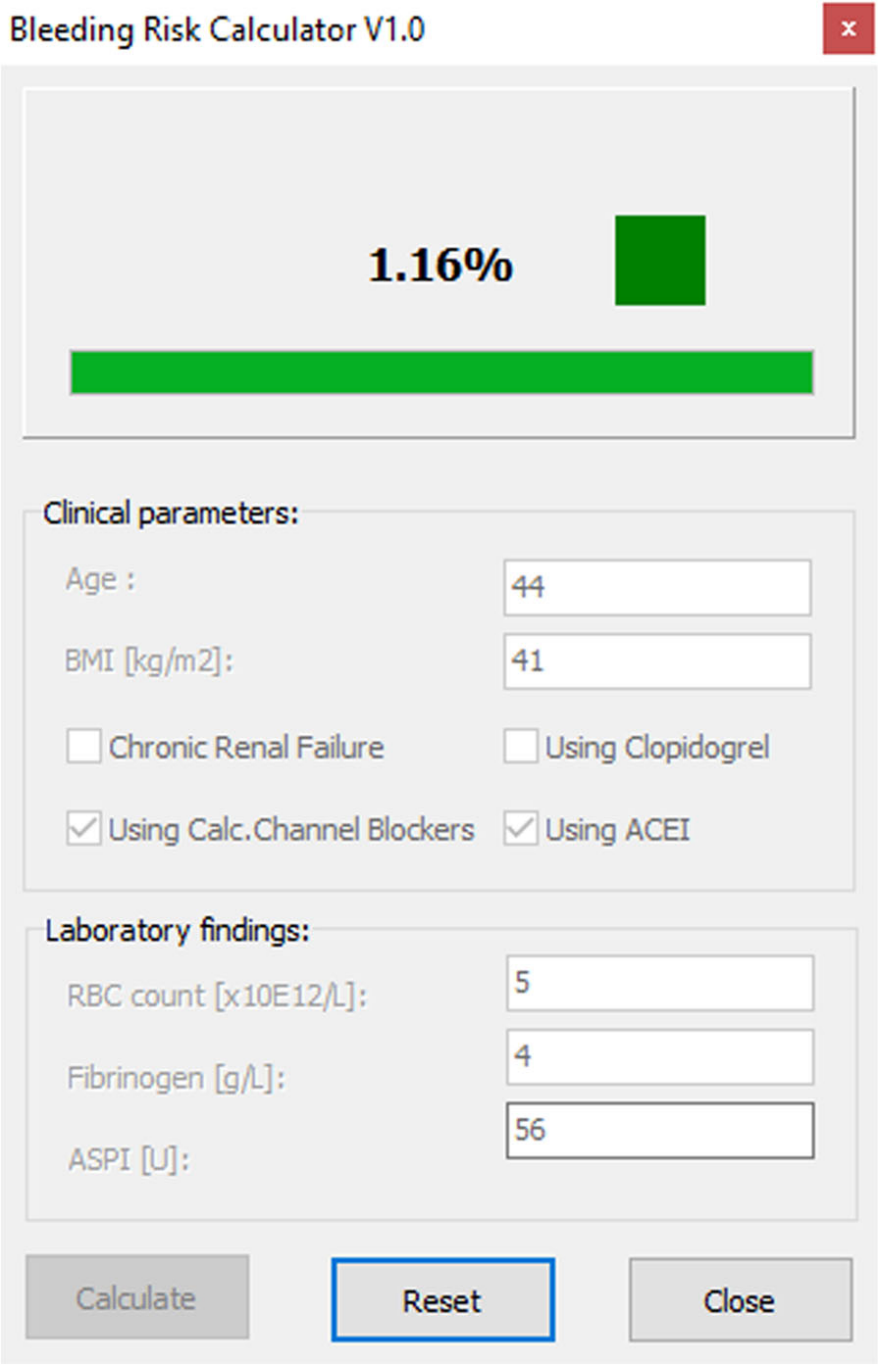
Case scenario 2

A 44 year old patient is scheduled for CABG to treat triple vessel disease which was recently diagnosed. The patient’s BMI is 41 and has hypertension for which he takes ACE inhibitors and calcium channel blockers. RBC 5 × 10^12^ and Fibrinogen 4 g/L was present in the lab findings. The patient was not exposed to any antiplatelet drugs and his Multiplate® ASPI test value is 56 AUC units suggesting normal platelet function (Fig. 4). SHOULD-NOT-BLEED-SCORE calculates bleeding risk to be 1.16% coupled with a green colored square suggesting low risk of bleeding.

### Case scenario 2 interpretation

This patient has a low risk of excessive bleeding. All demographic and laboratory findings suggest that the patient should not bleed. This is an example of a patient where transfusion treatment should be avoided. Of course, this patient may experience excessive bleeding, in that case, we should direct bleeding management towards surgical management (i.e. a surgical cause of bleeding).

## Discussion

A number of scoring methods are available for bleeding risk prediction in adult cardiac surgery [1, 12–16]. However, all but the WILL-BLEED risk score [1] are nonspecific addressing general adult cardiac surgery cases.

CABG remains one of the most commonly performed major surgeries, with well-established symptomatic and prognostic benefits in patients with multivessel and left main coronary artery disease [17]. CABG is on average performed at a rate of 44 per 100,000 individuals [17]. Number of CABG procedures per 100.000 inhabitants varies widely from 4 per 100,000 in Mexico to 79 per 100,000 in the United States and 91 per 100,000 in Hungary, respectively [17]. Reported transfusion rates for isolated CABG (rising over 90% with a huge proportion of those transfusions being unnecessary) call for a user-friendly screening tool to stratify bleeding risk and patients identified with low risk define the subgroup of patients to whom transfusion treatment might be avoided.

Several important considerations should be made when it comes to the development of the bleeding risk score:

1) Homogeneity of the study population makes it more reliable to create a scoring system. We know without a scoring system how complex cardiac surgery procedures carry a markedly higher risk of bleeding than isolated CABG.

2) The parameters we consider when thinking of bleeding risk constantly evolve. Some of those parameters are persistent, though. Our understanding, however, of bleeding risk evolves and when thinking of the inextricable association between bleeding and transfusion requirements, our focus switched from personalized point-of-care guided transfusion management in high-risk patients towards complete avoidance of transfusion in patients previously considered to have a low predicted risk of bleeding. Our SHOULD-NOT-BLEED bleeding risk score identifies patients at low risk of bleeding with a specificity as high as 94% and sensitivity of 24%.

Having in mind how transfusion rates climb up to over 90% in CABG patients, it becomes apparent that our target was to identify low bleeding risk CABG patients for whom transfusion treatment might be avoided.

### SHOULD-NOT-BLEED score compared to other bleeding risk scores

r The ROC analysis of the SHOULD-NOT-BLEED risk score calculator showed an adequate discriminatory ability (AUC 0.723 95% CI (0.694–0.753), *p* > 0.001). This discriminatory ability is comparable to the WILL-BLEED score (AUC 0.725, 95% CI 0.686–0.763, *p* = 0.033) [1]. It is important to stress how the SHOULD-NOT-BLEED score is designed to recognize patients at low risk for bleeding, whereas the WILL-BLEED, as well as other predictor scores including: the ACTION score [18], CRUSADE score [19], Papworth score [12], TRUST (Transfusion Risk Understanding Scoring Tool) score [13], and TRACK (Transfusion Risk And Clinical Knowledge) bleeding score [14], each of which were designed to identify patients at high risk of bleeding.

The WILL-BLEED bleeding risk score [1] was, to the best of our knowledge, the only score based on the isolated CABG population. The SHOULD-NOT-BLEED score is now the second one based on isolated CABG patients. Because patients undergoing off-pump CABG were excluded from the data analysis [8], The SHOULD-NOT-BLEED score may not be applicable for patients undergoing this surgical procedure. Such an approach sounds reasonable given the fact that on-pump CABG substantially differs to off-pump CABG (the use of cardiopulmonary bypass alters haemostatic mechanisms) as well as the priority given to study cohort homogeneity.

The problem with scores being developed on the general adult cardiac surgery population is that the scoring system inevitably carries non-specific parameters such as “complex cardiac surgery”. Moreover, it seems less feasible to use the same score for off-pump cases, on-pump CABG and complex aortic surgery cases. Therefore, our concept presents a kind of shift towards a precise, more focused, and personalized approach. Such an approach sets a priority to a homogeneity of the study cohort. The discriminatory ability of the SHOULD-NOT-BLEED score (AUC 0.723) is comparable to the WILL-BLEED score (AUC 0.725) [1]. In the WILL-BLEED score, few baseline characteristics and information on “potent” antiplatelet drugs use allows an accurate stratification of bleeding risk [1]. Our score presents a more accurate assessment of preoperative haemostatic properties. We have a parameter on recent (less then 5 days) clopidogrel use, which is more or less the case for the WILL-BLEED score. In contrast to our score, WILL-BLEED accounts for potent antiplatelet drugs use within 5 days. Our database of isolated elective CABG patients allowed only for the assessment of recent clopidogrel use. This is more specific to the drug evaluated in the context of bleeding risk. On the other hand, the chances to include elective patients exposed to ticagrelor for further studies are small as elective patients strictly adhere to the current guidelines on dual antiplatelet therapy. The SHOULD-NOT-BLEED score provides a more detailed insight into haemostatic properties. Our application is based on a single center database where all patients were exposed to Aspirin preoperatively and Aspirin was continued throughout the procedure. Accordingly, there was no need to specify whether or not someone was using Aspirin preoperatively into the app. However, our research group previously confirmed the presence of a subset of patients who have a prolonged and pronounced platelet inhibitory response to Aspirin [20], which in turn reflects bleeding tendency. We recently showed that patients with an adequate platelet inhibitory response to Aspirin are prone to excessive bleeding [20]. A Multiplate ASPI test value of AUC < 25 U was found to be predictive of excessive bleeding (OR 2.82 [95% CI 1.43–5.55], *p* = 0.003) which generates the idea of a subset of patients who have pronounced platelet inhibition on Aspirin therapy and who could benefit from preoperative Aspirin cessation [20]. The mathematical risk modelling platform used for the SHOULD-NOT-BLEED risk score has also proved that the Multiplate ASPI test value is as an independent predictor for bleeding [8]. The SHOULD-NOT-BLEED risk score is the first bleeding risk score that accounts for drug specific platelet reactivity in calculating bleeding risk. Current guidelines on dual antiplatelet therapy suggest continuation of Aspirin peri-procedurally [21]. Our approach adds to current knowledge and will hopefully contribute to the change of this paradigm. It is apparent that some patients under Aspirin treatment have a higher risk of bleeding (OR 2.82), therefore, inclusion of an Aspirin sensitive platelet function test into the bleeding risk score calculator sets a new moment.

The homogeneity of the study cohort rules out some of the confounding variables and leaves space for some new predictors. The WILL-BLEED score was designed to detect patients undergoing CABG who are at high risk of bleeding and to modify antithrombotic treatment if possible [1]. In other words, the WILL-BLEED score was mainly driven by the idea that a proportion of patients undergoing CABG are at high risk of bleeding and as such may be subject to possible haemostatic interventions, be it pre and/or intraoperative intervention. In contrast, our SHOULD-NOT-BLEED score is driven by the idea that a huge amount of transfusions (up to 93% according to the literature) in patients undergoing cardiosurgical procedures such as CABG which carry the lowest risk of bleeding are, in fact, unnecessary. The first step in addressing unnecessary transfusions in patients undergoing CABG is to identify patients primarily considered to be at low risk of bleeding. Our paradigm is that all patients with a high risk of bleeding should be treated in the same/similar way using point-of-care (POC) -guided transfusion algorithms to optimize haemostasis. The major clinical and economic burden arises from unnecessary transfusions, and when it comes to unnecessary transfusions, we should start with patients undergoing low risk procedures such as CABG who are at the same time at a low risk of bleeding.

Another advantage of the SHOULD-NOT-BLEED score is that use of the application is user friendly and self-explanatory. The idea of this application is not to guide and/or alter the clinical decision-making process. Since the development of this application is based on and validated by data from a primary source, it may be a useful tool for clinicians involved in preoperative screening and risk assessment.

Being based on the data from elective patients and primarily focused on elective patients makes this application not just a useful tool for preoperative risk assessment, but it also allows for modifiable parameter modification prior to surgery. For example, if someone is using clopidogrel in close proximity to surgery and at the same time has a low ASPI test value, postponing surgery with temporary discontinuation of Aspirin could modify the risk of bleeding.

The same holds for anemic patients with low a red blood cell count before surgery. The optimization of the red blood cell mass is the first pillar of patient blood management and may easily be considered modifiable if it contributes to high bleeding risk before surgery, as per the SHOULD-NOT-BLEED application. On the other hand, a low fibrinogen level contributing to high risk of bleeding on the calculator may prompt early fibrinogen supplementation if bleeding occurs after surgery.

When the statistical model used to design the SHOULD-NOT-BLEED-SCORE was applied to our existing database, an astonishing reduction of 39.1% in transfusion requirements could theoretically be reached. The cost savings reach 48.2% for PRBCs, 38.9% for fresh frozen plasma (FFP), 10.9% for platelet concentrate and 17.9% for fibrinogen, respectively [8]. Aforementioned cost savings pertain solely to blood product manufacturing costs [8]. Having in mind the additional cost of product administration as well as overhead expenses and the costs associated with treating complications secondary to transfusion therapy itself, it becomes apparent that real-life cost savings could potentially be much higher. Notably, indirect costs of transfusion treatment may reach over 65% of all expenditures related to transfusion therapy [22, 23].

The SHOULD-NOT-BLEED bleeding risk score may serve as an impetus for further refinements in haemostatic management. For this reason, we call for multicentric collaboration in developing and refining haemostatic management. Firstly, we propose validation of this score through multicenter collaboration. Secondly, cost-effectiveness of such a score may be calculated in a stepped wedge design prospective interventional multicentric trial [23]. Multicenter collaboration would yield a huge database allowing for more complex statistics. More patients recruited would make it possible to add some new parameters in considerations. We know from our practice several factors that could be implemented into considerations and this could be achieved in new study with bigger study cohort. We need to count on more parameters contributing to the bleeding risk. On the other hand, the major limitation of the current scoring system is that all parameters need to be available so to calculate the bleeding risk. The next generation of the bleeding risk score should be able to calculate the risk even in cases where missing values for some parameters. For this new generation risk scoring app, we need multicentric collaboration in order to recruit more patients and to achieve study sample size that would allow for such analyses.

## Conclusion

In conclusion, the SHOULD-NOT-BLEED-SCORE bleeding risk stratification application seems to be a simple tool to stratify patients according to bleeding risk with the focus of identifying patients undergoing CABG who are at low risk for excessive bleeding. Furthermore, our application represents a step forward as it may be readily available bedside, be it in a preadmission clinic, preadmission bay or operating theatre. We proved that this concept may reduce unnecessary transfusions and avoid a significant economic burden associated with unnecessary transfusions [8].

## Data Availability

The datasets during and/or analyzed during the current study are available from the corresponding author upon reasonable request.

## Abbreviations

AUC: Area under the curve
BMI: Body mass index
CABG: Coronary artery bypass grafting
FFP: Fresh frozen plasma
ICU: Intensive care unit
POC: Point of care
US: United States
